# Evaluation of Superficial and Dimensional Quality Features in Metallic Micro-Channels Manufactured by Micro-End-Milling

**DOI:** 10.3390/ma6041434

**Published:** 2013-04-03

**Authors:** Karla P. Monroy-Vázquez, Aldo Attanasio, Elisabetta Ceretti, Héctor R. Siller, Nicolás J. Hendrichs-Troeglen, Claudio Giardini

**Affiliations:** 1 Tecnológico de Monterrey, Eugenio Garza Sada 2501 Sur, Monterrey, NL 64849, Mexico; E-Mails: monroy.karla@gmail.com (K.P.M.V.); nicolas.hendrichs@itesm.mx (N.J.H.T.); 2 Department of Industrial and Mechanical Engineering, University of Brescia, Via Branze, Brescia 38-25123, Italy; E-Mails: attanasi@ing.unibs.it (A.A.); elisabetta.ceretti@ing.unibs.it (E.C.); 3 Department of Engineering, University of Bergamo, Via Marconi, Dalmine 5-24044, Italy; E-Mail: claudio.giardini@unibg.it

**Keywords:** micro-milling, micro-channels, fuel cells, heat exchangers, micro-fluidic devices

## Abstract

Miniaturization encourages the development of new manufacturing processes capable of fabricating features, like micro-channels, in order to use them for different applications, such as in fuel cells, heat exchangers, microfluidic devices and micro-electromechanical systems (MEMS). Many studies have been conducted on heat and fluid transfer in micro-channels, and they appeared significantly deviated from conventional theory, due to measurement errors and fabrication methods. The present research, in order to deal with this opportunity, is focused on a set of experiments in the micro-milling of channels made of aluminum, titanium alloys and stainless steel, varying parameters, such as spindle speed, depth of cut per pass (*a*_p_), channel depth (*d*), feed per tooth (*f*_z_) and coolant application. The experimental results were analyzed in terms of dimensional error, channel profile shape deviation from rectangular and surface quality (burr and roughness). The micro-milling process was capable of offering quality features required on the micro-channeled devices. Critical phenomena, like run-out, ploughing, minimum chip thickness and tool wear, were encountered as an explanation for the deviations in shape and for the surface quality of the micro-channels. The application of coolant and a low depth of cut per pass were significant to obtain better superficial quality features and a smaller dimensional error. In conclusion, the integration of superficial and geometrical features on the study of the quality of micro-channeled devices made of different metallic materials contributes to the understanding of the impact of calibrated cutting conditions in MEMS applications.

## 1. Introduction

In recent years, with the rapid progress in micro-electromechanical systems (MEMS), many micromachining methods have been developed to satisfy the increasing demand for fast, direct and mass manufacturing of meso- (100 μm–10 mm)/micro- (0.1–100 μm) devices, with high aspect ratios and superior surfaces [1,2]. The motivation for micro-manufacturing has been the same since manufacturing was established: better performance, lower-cost and high quality in order to use these devices for new and different applications, such as in fuel cells, heat exchangers and microfluidic devices [3,4].

A heat exchanger is equipment built for efficient heat transfer from one medium to another. The interest in the use of micro-channel heat exchangers (MCHE) has arisen, as they play an important part in the field of energy, as they can tolerate higher operating pressure, providing a larger surface area per unit volume, which results in a more efficient heat transfer and, moreover, its smaller size. The compactness in a MCHE is enabled by an array of micro-channels, which improves the heat transfer coefficient, which varies inversely with the width of the channels, and therefore, there exists a cost reduction advantage compared to the regular tube heat exchangers, as this minimizes the size and material used in manufacturing, such as the refrigerant needed on the system [5,6].

Meanwhile, fuel cells are devices made up of three segments, which interact together: anode, electrolyte and cathode. The results of these interactions are two reactions: fuel consumption and the creation of water/carbon dioxide, along with an electric current. Fuel cells require a constant source of fuel and oxygen to run, but they can produce electricity as long as these inputs are supplied; consequently, they have been proposed as a possible power source to address issues that involve energy production, enabling portable energy sources. It has been shown that microfabrication technology can be effectively applied for the miniaturization of fuel cells through the application of microchannel technology, allowing them to be small, efficient, modular and potentially inexpensive nowadays [7]. Many researchers are currently developing microchannel heat-exchangers, reactors and separators as components for compact hydrogen generators for fuel cells [8].

**Figure 1:**
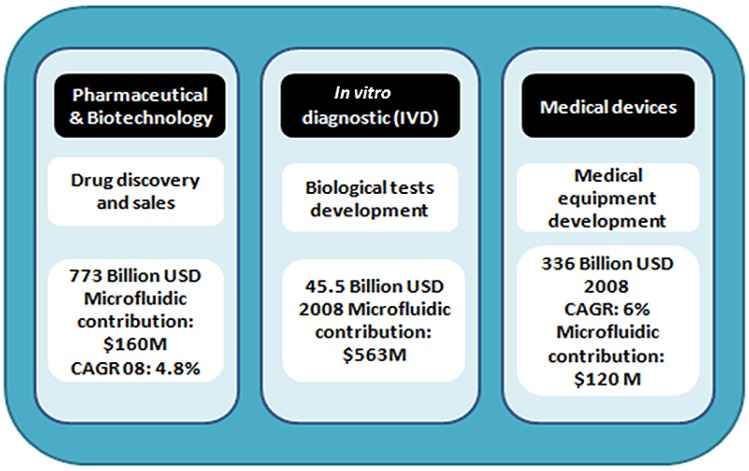
Microfluidic market considerations [9].

On the other hand, a microfluidic device is defined as an engineered component designed for the management and/or analysis of very small volumes of fluids. These devices can be identified by the fact that they have one or more channels with at least one dimension less than 1 mm. Therefore, the micro-channels forming the microfluidic device are connected together so as to achieve a desired function (mix, pump, redirect or allow chemical reactions in a cell). The miniaturization of fluidic devices offers several practical benefits, such as using a low sample volume, occupying little space and achieving high throughput. Moreover, microfluidic devices are now developing several applications in areas like medicine and biotechnology for the separation of molecules, transportation of DNA and drug delivery systems (Figure 1) [9].

The latter devices were enabled with the micro-channel design, whose working principle is evaluated by measures of heat transfer and fluid flow rates. However, studies denoted significantly deviated results from conventional theory on both flow rates, which apparently are due to measurement errors and fabrication methods, including the surface roughness and the channel shape. Surface roughness is considered to be one of the most important factors for these deviations, because when the channel dimension reduces, the importance of roughness increases [5,10,11,12,13]. Although the effect of surface roughness on the flow has been well studied for decades, routinely, the products are not able to control the roughness of surfaces to the threshold levels [8,14]. Past experimental studies on liquid flow in circular, rectangular and trapezoidal micro-channels showed that roughness and geometric deviations play an important role in the performance of fluid flow, as it affects the pressure drop through a microchannel and increases the friction factor more than the heat transfer coefficient occurring from the transition from laminar to turbulent flow. Therefore, it should be at most avoided during the fabrication of microchannels used in heat transfer and fluid-based technologies [5,15,16,17,18].

**Figure 2:**
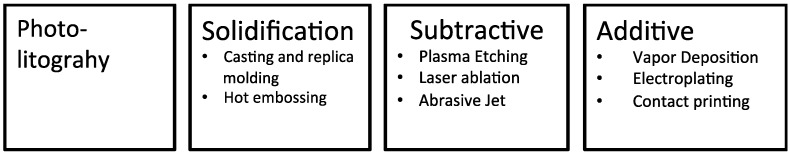
Broad categories of micro-fabrication processes for micro-channels [19].

Micro-channels have largely been triggered by advances in microfabrication technologies, which currently present difficulties related to the behavior of the tool, material and cutting mechanism, as in the micro-scale, it becomes a crucial phenomenon that can have a direct impact on the compliance of these features with an acceptable quality (surface finish, dimensional accuracy and fluid-heat flows). As a response to the actual demands, micro-manufacturing processes (shown in Figure 2), such as lithography, casting, micro-machining and additive technologies, have emerged and been documented in the literature [19]. Although micro-manufacturing has been discussed abundantly, research dealing with the manufacturing methods for biomedical components, micro-turbines and pumps for fuel cells and heat exchangers are hardly found, suggesting that the current knowledge is still not enough to deal with the inaccessibility, high cost or time consumption in the production of actual microcomponents [20,21].

Lithography, among the micro-manufacturing technologies, is the conventional process for micro-channels, as it shows good surface and topographic quality, although micro-milling is now achieving contending levels in performance evaluation in terms of product quality, surface finish and savings potential [22]. Consequently, processes, such as micro-end-milling, are in full expansion, as they can be produced by a cost-efficient approach along with good accuracy, low surface roughness and high material removal rates (MRR) in micro-features as small as 5–10 μm [4]. It appears, then, that micro-milling can be an ideal candidate to produce micro-forms in a flexible and a fast way for these applications.

Therefore, the present article is intended to conduct a micro-milling experiment on metallic alloys in order to analyze the results in terms of surface quality and dimensional features to evaluate its performance as a candidate technology for the prototyping of micro-channels. As a result, this work will also contribute to the understanding of the phenomena involved in this operation and the relations between process parameters and the quality of the geometrical final micro-features of the channel with micro-milling.

## 2. Experimental Section

**Figure 3:**
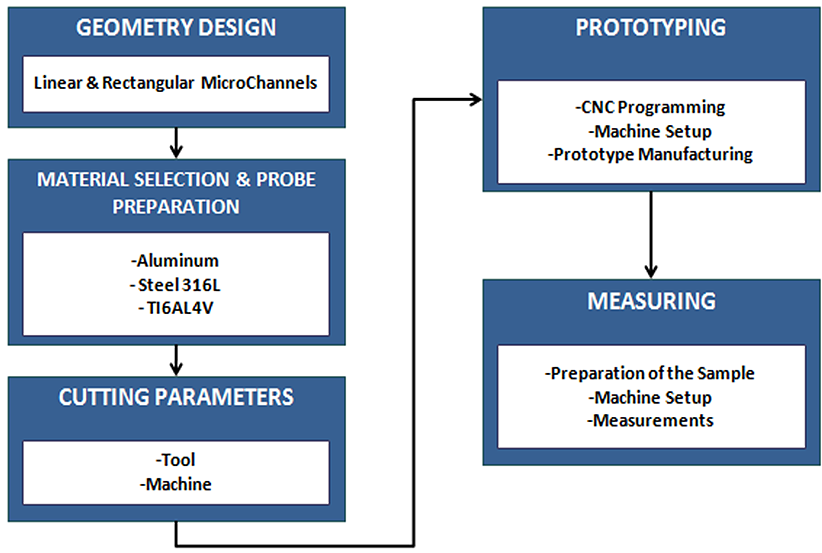
Experimental process of micro-milling manufacturing of micro-channels.

This section presents the experimental setup, measurement equipment and the overall methodology used to evaluate the average roughness of the channels, the dimensional measurements and the burr appraisal recorded in the micro-milling of channels. Simultaneously, the factorial experimental design and statistical methodology to analyze the measurements acquired in roughness, channels dimension and burr evaluation are presented. The steps followed to make the metallic micro-channels are represented in Figure 3.

### 2.1. Micro-Channel Design

**Figure 4:**
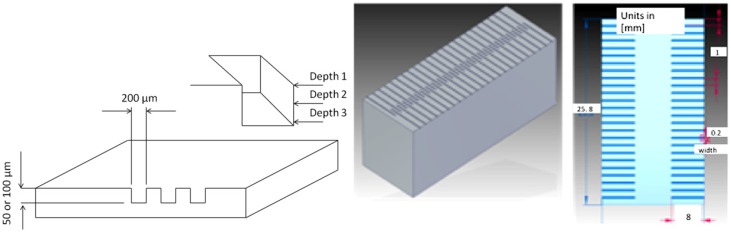
Micro-channel design.

The design of the planned geometry was a rectangular profiled micro-channel with 200 μm in width, 8 mm in length and in two different depths (50 and 100 μm). The Solid Edge software was used for CAD design of the micro channel geometry, and G-code was employed for programming the linear tool paths of the design through a parametric function, in an arrangement of 1 mm between each channel (Figure 4).

### 2.2. Experimental Plan

The design of experiments was defined as a two-level half fractioned factorial with five variable factors (spindle speed, depth of cut per pass, channel depth and feed per tooth, in dry and wet conditions). Consequently, a V resolution study (2_V_^4^) with three replicas was applied, totaling 48 micro-channels per material. The runs of the design of experiments have been sorted in a random manner; however, the same order of runs was used on the replicas in order to have comparable results for issues of tool wear. Therefore, a total number of one hundred and forty-four micro-channels have been machined by the experimental plan shown in Table 1 in which the variable factors and the levels are summarized. The stated cutting parameters have been selected from a preliminary experimental scheme [23] that will permit the outcome to be comparable and the milling center performance to stand out also, as the machine used in the present research had better specifications for micro-machining.

**Table 1:** Variable factors and levels of milling experimentation.

Variable Factor	Aluminum		Titanium/Steel	
	L1	L2	L1	L2
Spindle speed [*S*], min^−1^	10,000	12,000	10,000	12,000
Depth of cut per pass [*a*_p_] µm	2	10	2	10
Channel depth [*d*], µm	50	100	50	100
Feed per tooth [*f*_z_], µm/fz	1.25	1.90	0.625	1.25
Coolant	Dry	Wet	Dry	Wet

### 2.3. Equipment and Material

**Figure 5:**
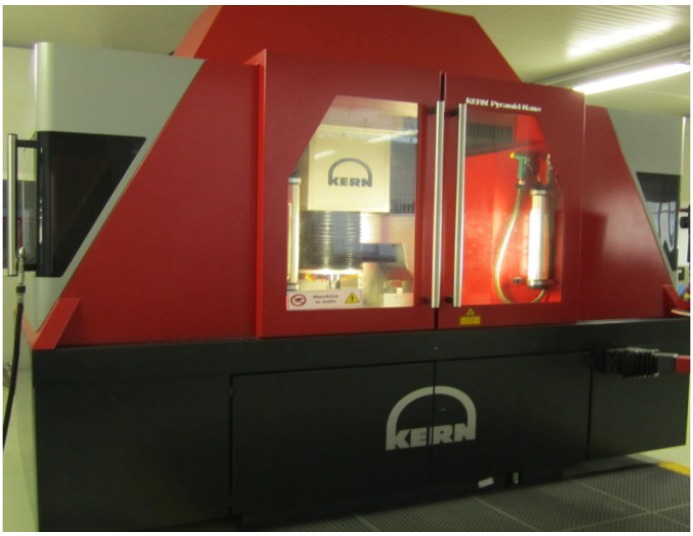
KERN pyramid nano-machining center.

**Figure 6:**
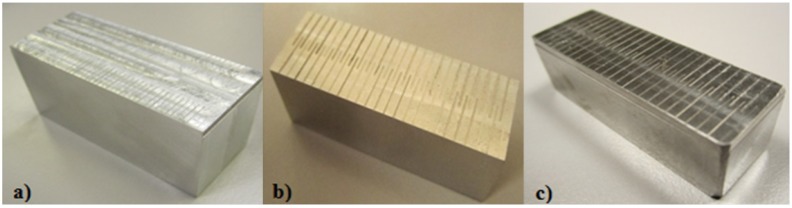
Workpiece probes: (**a**) Al alloy (56 mm × 23 mm); (**b**) AISI 316L (40 mm × 14 mm); (**c**) Ti_6_Al_4_V (42 mm × 14 mm).

These geometric elements (micro-channels) were machined on a five-axis CNC machining center with a vertical spindle and a Heidenhain iTNC 530 Controller, which is shown in Figure 5. The experimental sample materials used in the present study were metal alloys, such as aluminum (hardness value of 21 hardness Rockwell B-scale (HRB)), stainless steel 316L (AISI 316L) and Ti_6_Al_4_V. These workpieces were then cut into rectangular probes for the micro-milling process (Figure 6).

Table 2 shows the geometric characteristics of a 200 μm diameter end-mill (Mitsubishi^©^ MS2SSD0020), which was used to machine the rectangular micro-channels. The same end-mill was employed for all 16 channels; therefore, three identical end mills were used for each material.

**Figure 7:**
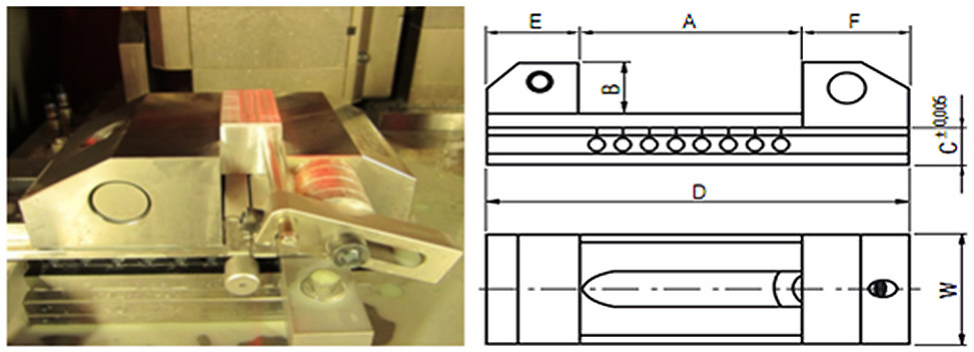
Set up on KERN clamp.

The workpieces were fixed up in a standard clamp Gerardi vise series (Figure 7), as the workpieces were rectangular; consequently, a special fixture was not required.

**Table 2:** Tool geometric characteristics.

Mitsubishi^©^ MS2SSD0020	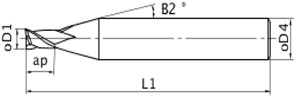
Material/coating:	Solid carbide/CRN
Number of flutes (*z*):	2
Cutting diameter (D1) [mm]	0.2
M ax. cutting edge diameter tolerance	0
Min. cutting edge diameter tolerance	−0.02
Shank diameter (D4) [mm]	4
Overall length (L1) [mm]	40
Length of cut (*a*_p_) [mm]	0.3
Tool interference corner (B2) [°]	15

### 2.4. Metrology and Sample Preparation

The response variables were surface roughness in *R*_a_ [μm] at the floor of the microchannel, width of the channel in [μm], depth of the channel in [μm] and the micro-channel profile form. In Table 3, several metrology procedures are presented that were executed in order to measure the response variables stated previously.

**Table 3:** Metrology response variables.

Response Variable	Measurement Type	Evaluation
Burr formation	Qualitative	Rating 1–5
Shape	Qualitative	Profile
Dimension	Quantitative	Width size [μm]
Roughness	Quantitative	*R*_a_ [μm]

**Figure 8:**
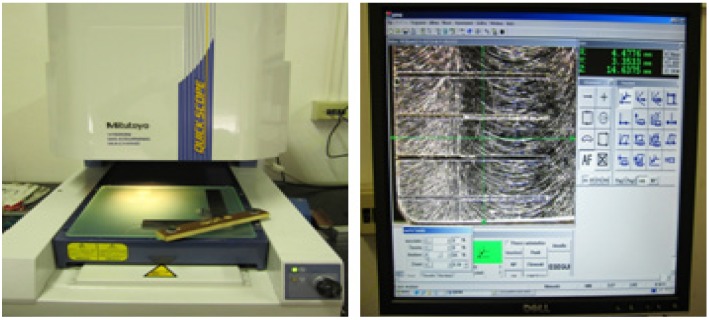
Mitutoyo QuickScope QS200Z & QSPAK software.

**Figure 9:**
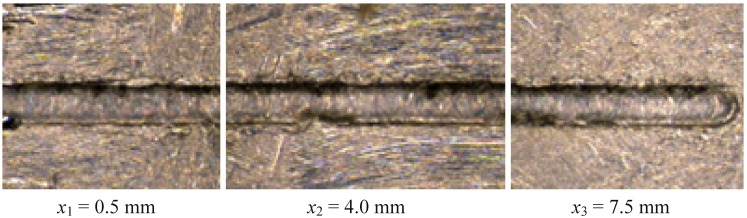
Channel burr formation map. *x* *=* micro-channel length.

Initially, a burr evaluation was made in a qualitative approach from images acquired with a non-contact vision measuring machine; Mitutoyo Quick Scope QS200Z along with QSPAK software for the compilation of images (Figure 8). Three images were taken from every channel, varying the position of the channel. Consequently, snapshots at 0.5 mm, 4.0 mm and 7.5 mm were taken, and a complete map of the top burr could be formed (shown in Figure 9), and afterward, it was evaluated in a given scale from 1 to 5, 5 being no burr and 1 excessive burr.

**Figure 10:**
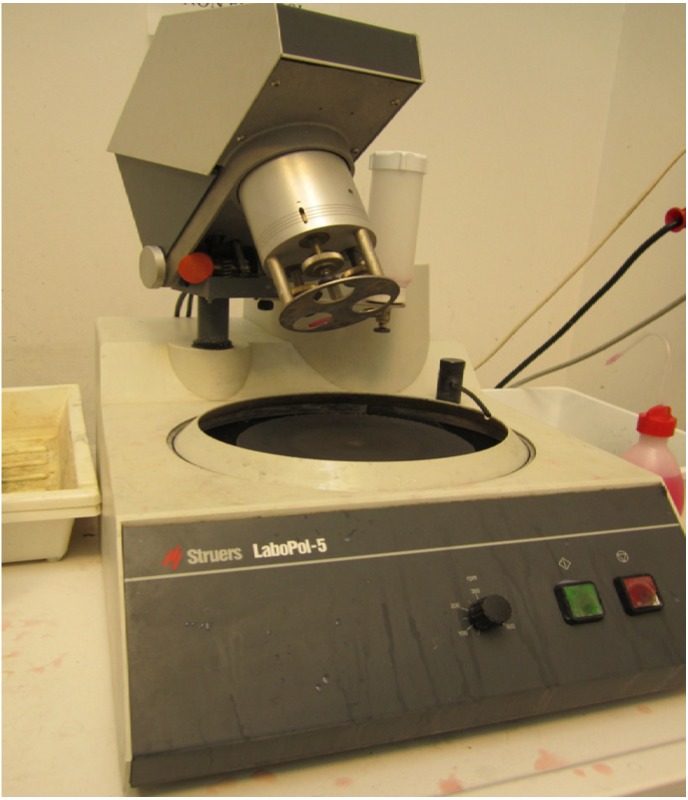
Struers polishing machine.

A sample preparation was made by standard metallographic means (Table 4) in a Struers polishing machine (Figure 10) in order to assure the correct gathering of information, discarding the noise effects of burr and chip building at the entrance of the channel.

**Figure 11:**
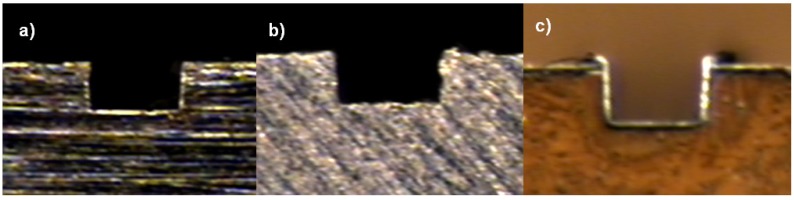
Profiles shapes (**a**) Ti_6_Al_4_V; (**b**) AISI 316L and (**c**) Aluminum alloy.

The channel profile was then measured through image capturing, and the analysis was performed in a qualitative way, trying to categorize the final shapes derived by the process in each material (Figure 11).

**Figure 12:**
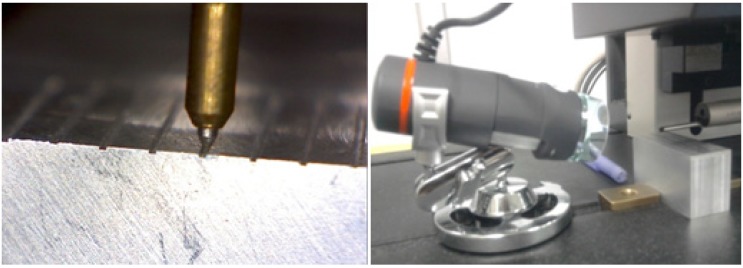
Channel alignment by Celestron portable microscope.

Moreover, the dimensional analysis was executed via micrographs. In order to help the shape analysis and to record the deviation in reference to the nominal width channel size, measurements of the channel size were carried out in three different depth levels, as in Figure 4. By the counterpart, surface roughness (*R*_a_) measurements from the floor surface of the micro-channel were conducted by a profilometer Zeiss SURFCOM 1500SD2, with a cut off of *λ*_c_ = 0.8 mm and a sampling of *l*_s_ = 4.0 mm, in accordance with ISO/DIS4287/1E, using a Gaussian profile filter and no tilt correction. The accuracy of the roughness measurements was defined by a faultless alignment of the channel path with the *x*-axis movement of the stylus on the profilometer. Consequently, the alignment was possible by the aid of a Celestron 44302-A handheld digital microscope, which permitted the correct monitoring through video and magnified image capturing of the stylus in the center of the microchannel (shown in Figure 12), and then, avoiding the friction of the stylus within the channel walls, which itself can be derived in biased measurements.

**Table 4:** Metallographic specifications.

Material	SiC Grain Size	Cloth Size [μm]
Aluminum alloy	80-220-500-800-1200	1
Stainless steel 316L	80-120-220-320-500-800-1000	1–3
Ti_6_Al_4_V	320-500-800	1

### 2.5. Statistical Analysis

The obtained data was statistically studied by analysis of variance (ANOVA) to quantify the effect of the process parameters: spindle speed [*S*], depth of cut per pass [*a*_p_], channel depth [*d*], feed per tooth [*f*_z_] and coolant and its interaction on the response variables presented in Table 3.

### 2.6. Measurement Systems Validation

A gauge repeatability and reproducibility (R&R) test was carried out on the profilometer for data validation. Therefore, roughness measurements were taken from three channels, 15-times each, where the results showed sufficient different categories to distinguish adequately between parts and a low 7.09% of repeatability, which means, according to the AIAG guidelines, the measurement device is acceptable.

## 3. Results

The experimental output was a total of 144 micro-channels, 48 per material, following the methodology presented in Section 2. The gathered results were analyzed by material and by observing the mean average roughness, mean burr formation evaluation, channel mean width and its percentage error deviation relative to the nominal size, channel depth and its percentage error deviation relative to the nominal size (50 or 100 µm) and the predominant profile shape resulting from the process.

### 3.1. Aluminum Alloy

**Figure 13:**
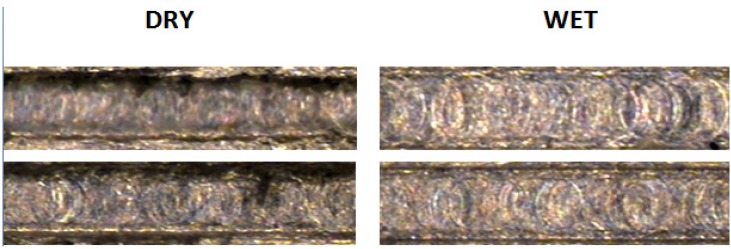
Top view of micro-channel showing roughness optimization by coolant application.

The surface finish was evaluated through a roughness measurement (*R*_a_), which showed considerable variations in the data acquired, the results ranging from 0.095 to 2.5 μm. By statistical analysis, it was found that the coolant factor has the most influence, as its usage considerably diminishes the roughness in the floor of the channel, as can be seen in Figure 13.

**Figure 14:**
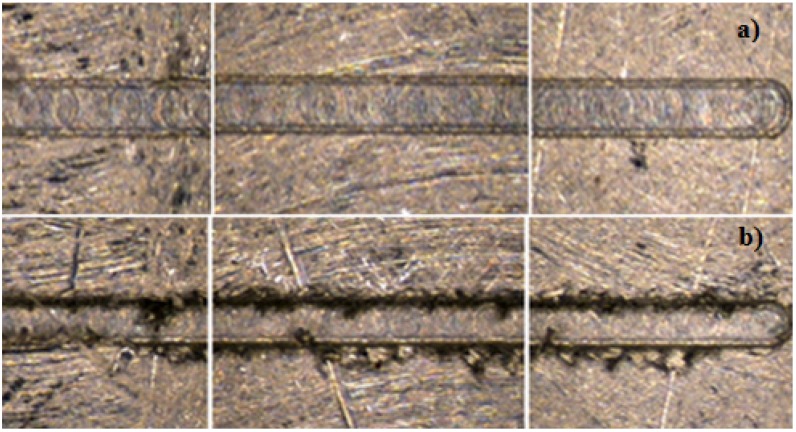
Coolant and *a*_p_ influence in burr formation on Al Alloy. (**a**) Low *a*_p_ with coolant and (**b**) high *a*_p_ and coolant.

The mean burr was evaluated as 3.10 out of five, this being the depth of cut per pass and the coolant application being the most important factor to consider in order to minimize the top burr formation of the micro-channels (Figure 14). By observing the two-way interactions of the depth of cut per pass and the coolant *versus* all other factors in each case, it was found that the minimal burr formation is achieved by the combination of the lowest level of depth of cut per pass (2 μm) and the use of coolant.

**Figure 15:**
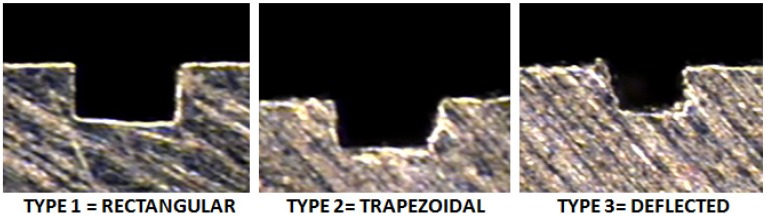
Common categorized profiles in Al alloy.

In the dimensional feature, a tendency toward a larger dimension on the width at the top level compared to the mid- and bottom of the channel was found, recurrently performing a trapezoidal shape (shown in Figure 15), which was proven to be statistically significant. By performing an ANOVA analysis independently, it was identified that the width dimensions in all depth levels of the channel were affected by the depth of cut per pass, which may cause deflection on the tool and, therefore, produce irregular micro-channels. The errors associated with the dimensional study were 11.89% in width and 8.34% in channel depth.

### 3.2. Austenitic Stainless Steel (AISI 316L)

**Figure 16:**
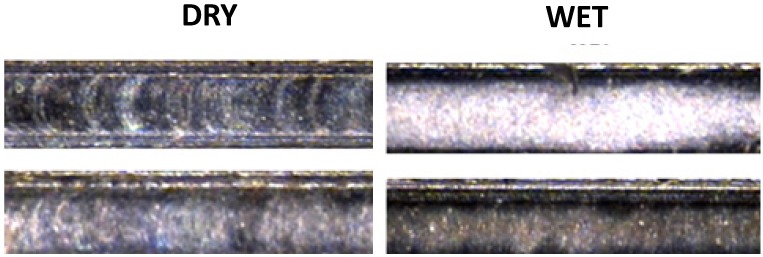
Coolant application for minimizing roughness in AISI 316L.

The roughness measurement results showed the best surface finishing of the three workpiece materials, ranging from 0.022 to 0.5840 μm. The ANOVA analysis gave evidence once more that the use of coolant reduces the roughness; therefore, wet conditions are favorable for optimizing the surface quality on the channel floor (Figure 16).

**Figure 17:**
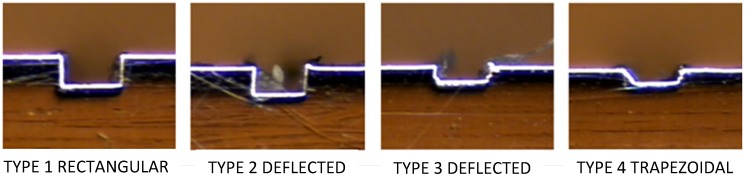
Common categorized profiles in AlSI 316L.

The burr formation was appraised in 3.31 out of five, the steel being materials the one that produced fewer burrs on the channel edges out of the three. The dimension measurement at three different levels on the channel (top, mid- and bottom) can give a basic explanation of the shape of the profile; by this, the propensity of performing a trapezoidal shape was found, as shown in Figure 17 (Type 4).

**Figure 18:**
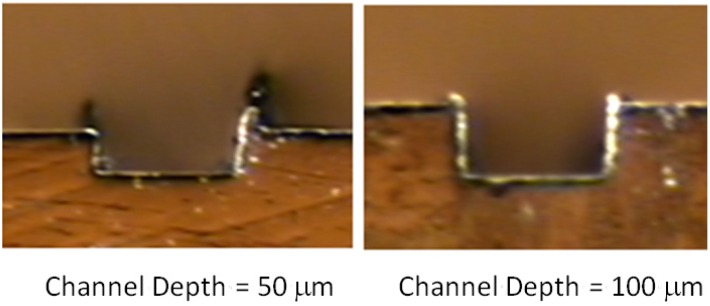
Channel depth influence on the shape deflection.

The dimensional errors were 4.98% and 11.80% in depth and width, respectively, giving, then, the most accurate dimensional depth measurements. By ANOVA test, it was found that the dimension of the channel was affected statistically in a significant quantity in the mid-level width size, in which the problem of deflection was more visible in low depth channels (50 μm). In Figure 18, the deformation presented on the material in the 50 μm channels can be seen. In this case, the channel width measurements always presented a smaller width size than the nominal radius of the tool, by which it can be supposed that the tool real diameter had to be smaller than the specified 200 μm.

**Figure 19:**
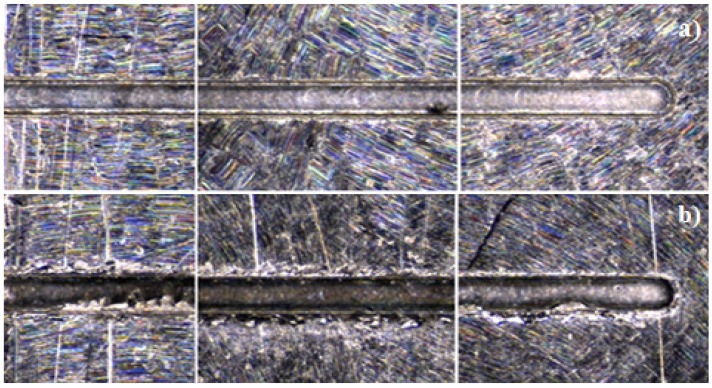
Depth of cut per pass influence in burr formation of AISI 316L. (**a**) Low *a*_p_ and (**b**) high *a*_p_.

The depth of cut per pass was the most influencing factor to minimize the top burr formation of the micro-channels (Figure 19), by which the least burr formation was obtained with the 2 μm level.

### 3.3. Titanium Alloy Ti_6_Al_4_V

**Figure 20:**
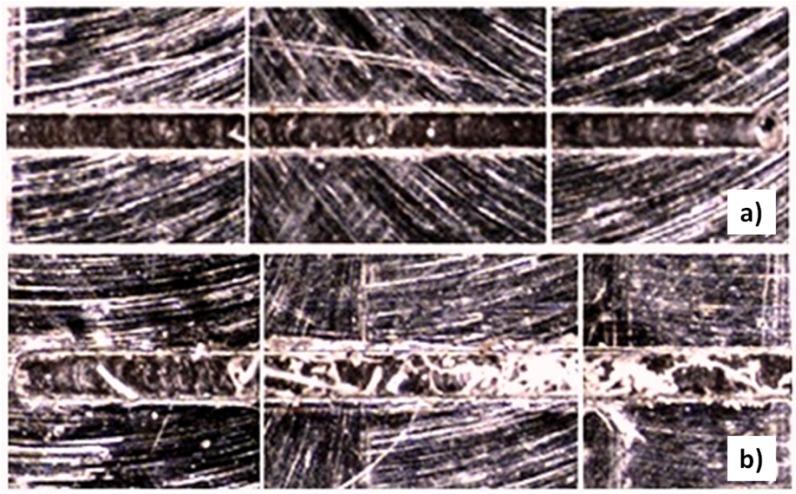
Depth of cut per pass influence in burr formation on Ti_6_Al_4_V. (**a**) Low *a*_p_ and (**b**) high *a*_p_.

**Figure 21:**
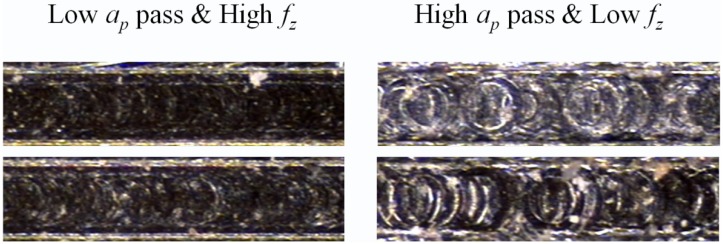
Depth of cut per pass (*a*_p_) and feed influence on the surface roughness of Ti_6_Al_4_V.

The roughness results presented a range from 0.043 to 0.574 μm, which indicates a good surface finish and lower dispersion in the outcome measurements. The burr evaluation was scored as 3.125; this case presented the same trend as aluminum and steel by the depth of cut per pass minimizing the top burr formation of the micro-channels being the main factor (Figure 20), specifically at 2 μm in depth of cut per pass. The two-way interaction analysis demonstrated that a low depth of cut per pass accompanied by a high feed per tooth presented the lowest roughness measurements (Figure 21).

Additionally, the top burr formation could also be described by tool wear, as can be seen on time series graphs in which the burr appears to trend negatively as the use of the tool increases.

**Figure 22:**
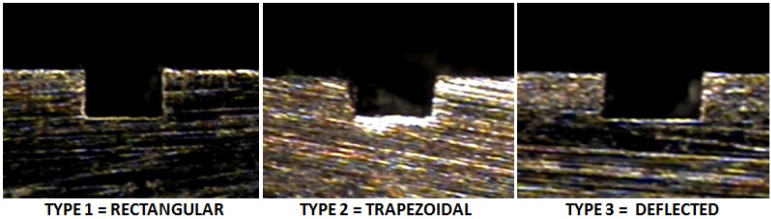
Common categorized profiles in Ti_6_Al_4_V.

In the evaluation of the titanium workpiece, a statistical difference was found only on the bottom level on the channel; the upper and middle level of the channel statistically satisfied the nominal measurement of 200 μm, the lower level of the channel being always the smallest dimension. Titanium was the material with the most regular profiled shape channels (See Figure 22).

The dimensional errors associated with the geometry deviation were 11.62% in depth and 5.33% in width. The titanium alloy reversed the error percentage trend, presenting the most accurate width measurements above all.

## 4. Discussion

According to the latter results, several phenomena in micro-cutting were observed, and the possible reasons and contrasting arguments with the literature are presented as follows:

(1) In the review presented by Camara *et al.* [21], discussions regarding tool geometry, tool materials, cutting parameters and other factors over burr formation and surface roughness in micro-machining of metallic materials are extensive, but there is scarce evidence of work regarding these effects and channel profiles in different metallic materials. The present work can contribute to a deeper understanding of the integrated effect of burr/surface roughness/geometry.
(2) All the materials’ top burr formation was affected by the depth of cut per pass. Lekkala *et al.* [24] realized a similar experiment on Al alloy and stainless steel in which the ANOVA analysis also showed the same trend on the significant influence of *a*_p_ on burr formation, proposing vibrations as the possible explanation. In contrast, in his study, stainless steel burrs appeared in larger amount than for aluminum. In the case of Ti_6_Al_4_V, the burr formation was minimized by a lower *a*_p_ (2 μm), contrasting a study of Schueler [25] in which by applying the same *a*_p_, massive burr formations occurred. The literature assures us that as the depth of the cut increases, the tool is pushed further beneath the surface of the work material; this would in turn impress the grooves on the surface being machined, because of the increased cutting load. Bisacco *et al.* [2] highlighted the ploughing phenomenon that could explain the behavior of titanium alloy *versus* burr formation, where a plastic deformation occurred when the depth of the cut was lower than a critical chip thickness. Kim *et al.* [26] classified the deformation into two types, the forced deflection of the tool and the elasto-plastic deformation of the workpiece material. The burr formation in all materials can also be explained by the tool wear, which is one of the most important aspects of machining. As in Lee *et al.* and Schmidt [27,28], the burr size in stainless steel was related to the amount of tool wear. If a built-up edge is encountered on the tool, the tool can continue to cut for a long time without wear, but this affects the channel dimensions.
(3) In Ti_6_Al_4_V, the roughness was minimized by a lower *a*_p_ (2 μm) and high feed rates (25 and 30 mm/min); this can explain that with a cutting depth so small and a high load, the cutting of the material does not exist any longer, but only plastic deformation and ploughing of the material. These results are supported by studies conducted by Yang and Chen [29], in which an increase on the depth of the cut that worsens surface roughness was encountered; these were also similar to Ginta *et al.* [30], which varied the cutting conditions in the same Ti alloy (Ti_6_Al_4_V). Korkut and Donertas [31] even stated a linear model, where the increase on the depth of cut increased the surface roughness.
(4) Run out was another issue with great impact in micro-machining. Due to the lower strength in the micro-tool, run out causes less stiffness and more vibrations than conventional processes, generating higher roughness or deflection of the tool. The deviation of the desired shape of the profiles can be due to this phenomenon creating a tool deflection, greatly affecting the chip formation and accuracy.
(5) According to Dornfeld [27], a typical flood of coolant is generally not suitable for micro-machining, because, first, the flow pressure may influence the cutting tool behavior and the removal of excess working fluid after micro-machining is challenging. However, in the present experiments, the presence of coolant was strongly proposed to minimize the roughness, as clearly seen in all three materials. Muthukrishnan *et al.* [32] show the influence of coolant in micro-milling of Ti_6_Al_4_V. Their results validated the present results, showing better roughness in wet machining, as coolant prevents the formation of a built up edge, which damages the surface.

## 5. Conclusions

In the present study, dimensional and geometric features in burr and surface roughness were evaluated as a result of a micro-milling process in metallic materials (aluminum, steel AISI 316L and Ti_6_Al_4_V). Results suggest that micro-milling offers a process capable of improving quality features required on the micro-channeled devices for a diversity of MEMS applications. The conclusive remarks can be summarized as follows:

(1) In general, a better surface quality in terms of burr formation and roughness was obtained in AISI 316L, although Ti_6_Al_4_V had the best behavior in forming the desired rectangular profile. The application of coolant was critical to get better quality superficial features, which avoided built up edge formation and heating of the material.
(2) In all three workpieces, burr formation can be controlled by a low depth of cut per pass. A low *a*_p_ (2 μm) minimizes burr formation in all materials, and it also influences accuracy in the dimensional measurements.
(3) The deviated shapes encountered were trapezoidal, regular and deflected. These profiles can be explained by the possible run out of the tool, tool wear and the built up edge.
(4) There is a general affinity in the machining process of developing V-shaped microchannels. Further research is needed for a better understanding of the behavior of the tool deflection in the three different materials, since this deviation affects heat and fluid flow.
(5) The critical phenomena encountered, like run-out, the ploughing effect, minimum chip thickness and tool wear, explained the deviations in the form, dimensions and surface quality, which can be minimized with a proper calibration of cutting conditions, mostly the depth of the cut, according to the findings of this work.
